# Supplementary material for: Development of SNP Markers from GWAS for Selecting Seed Coat and Aleurone Layers in Brown Rice (Oryza sativa L.)
Source: Genes (Basel). 2022 Oct 6;13(10):1805. doi: 10.3390/genes13101805 (PMC9602391; doi:10.3390/genes13101805)
Supplement: Supplementary file 1 [file genes-13-01805-s001.zip › genes-1831871-supplementary.pdf]

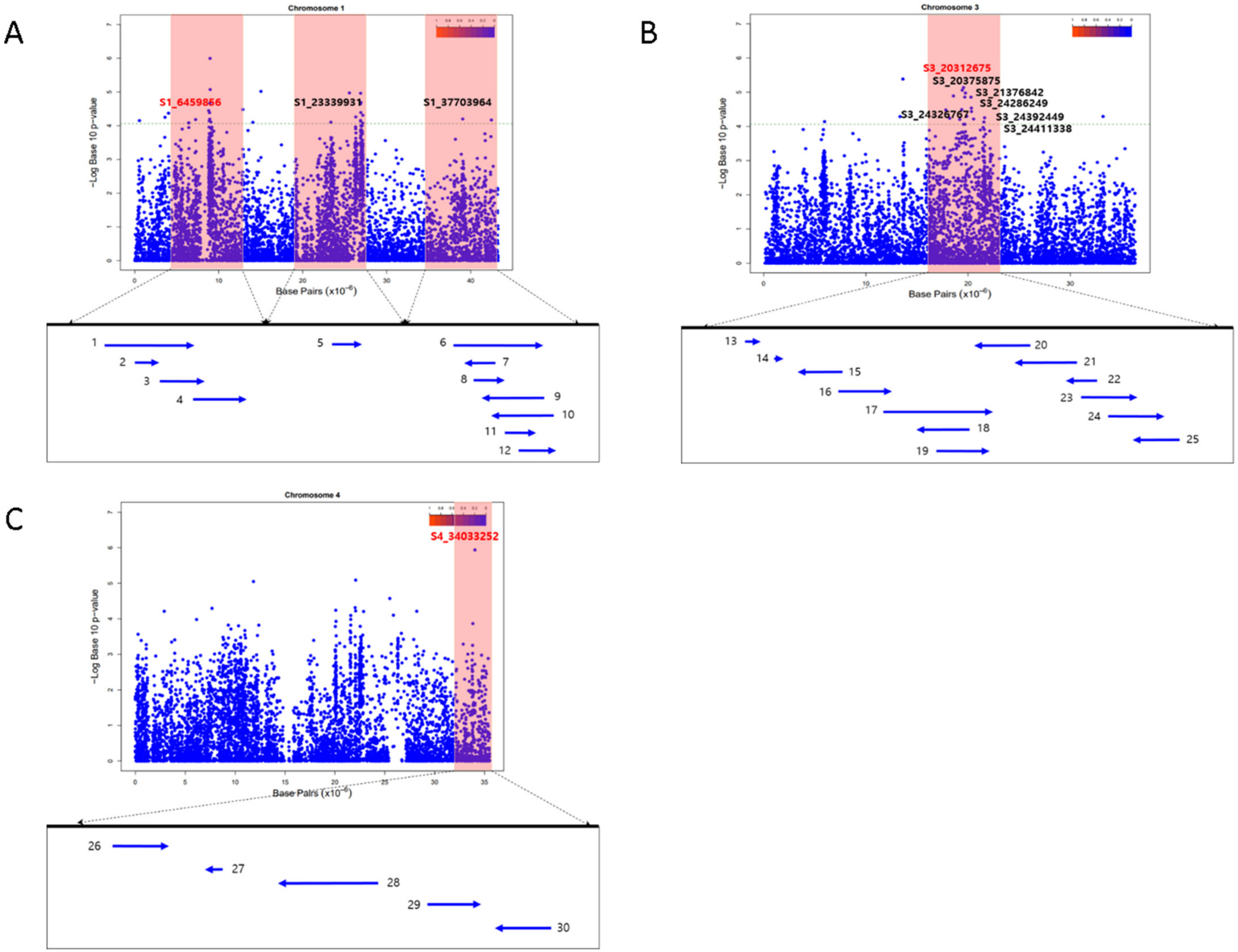

Supplementary Figure S1. Genome-wide Manhattan plots for significant SNPs within 100 kb at a signal GWAS locus. The names of genes corresponding to each number which from the region as highlighted in red are shown in Table 3. (A) Manhattan plots in chromosome 1, (B) Manhattan plots in chromosome 3, (C) Manhattan plots in chromosome 4.
